# Pp65 antigenemia, plasma real-time PCR and DBS test in symptomatic and asymptomatic cytomegalovirus congenitally infected newborns

**DOI:** 10.1186/1471-2334-10-24

**Published:** 2010-02-11

**Authors:** Sandro Binda, Antonella Mammoliti, Valeria Primache, Patrizia Didò, Carlo Corbetta, Fabio Mosca, Lorenza Pugni, Anna Bossi, Cristian Ricci, Maria Barbi

**Affiliations:** 1 Dipartimento di Sanità Pubblica - Microbiologia - Virologia, Università degli Studi di Milano, Milan, Italy; 2 Laboratorio di Riferimento Regionale per lo Screening Neonatale, AO ICP, Milan, Italy; 3 Istituto di Pediatria e Neonatologia, Fondazione IRCCS "Ospedale Maggiore Policlinico, Mangiagalli e Regina Elena", Università degli Studi di Milano, Milan, Italy; 4 Istituto di Statistica Medica e Biometria "GA Maccacaro", Università degli Studi di Milano, Milan, Italy

## Abstract

**Background:**

Many congenitally cytomegalovirus-infected (cCMV) neonates are at risk for severe consequences, even if they are asymptomatic at birth. The assessment of the viral load in neonatal blood could help in identifying the babies at risk of sequelae.

**Methods:**

In the present study, we elaborated the results obtained on blood samples collected in the first two weeks of life from 22 symptomatic and 48 asymptomatic newborns with cCMV diagnosed through urine testing. We evaluated the performances of two quantitative methods (pp65 antigenemia test and plasma Real-time PCR) and the semi-quantitative results of dried blood sample (DBS) test in the aim of identifying a valid method for measuring viral load.

**Results:**

Plasma qPCR and DBS tests were positive in 100% of cases, antigenemia in 81%. Only the latter test gave quantitatively different results in symptomatic versus asymptomatic children. qPCR values of 10^3 ^copies/ml were found in 52% of newborn. "Strong" DBS test positivity cases had higher median values of both pp65 positive PBL and DNA copies/ml than cases with a "weak" positivity.

**Conclusions:**

As expected antigenemia test was less sensitive than molecular tests and DBS test performed better on samples with higher rates of pp65 positive PBL and higher numbers of DNA copies/ml. The prognostic significance of the results of these tests will be evaluated on completion of the ongoing collection of follow-up data of these children.

## Background

Congenital cytomegalovirus infection (cCMV) is present in 0.7% of neonates [1] and represents one of the most feared events during pregnancy, causing not only immediate but also delayed sequelae to the infant. At birth, 10% of infected newborns present with symptoms involving the hematopoietic, hepatobiliary, or central nervous systems.

During early childhood, one fifth of all congenitally infected babies either die or suffer permanent neurological impairments that can affect normal development, particularly psychomotor retardation, ocular damage, or sensorineural hearing loss (SNHL). This last sequelae is the most frequent, occurring in about 20% of cases. It may be evident at birth (45-65%) [1] or develop later. Moreover, there may be different degrees of severity and it may be progressive.

As corrective measures may only be successful if carried out immediately, having diagnostic tools available to promptly identify an infected child is essential. This is most useful for children with late-onset SNHL, since the median age for detection of hearing loss is of 27 months (range, 25-62 months) [2].

It is also important to use virological markers that indicate an unfavorable prognosis to identify newborns at risk of developing serious problems. This forms the basis for organizing close follow-up, early rehabilitation, and/or therapy to minimize long-term damage.

The viral load in biological samples, such as urine and peripheral blood leukocytes (PBL) during the neonatal period was proposed by some investigators as a prognostic marker of developing hearing loss [3-6].

In this study we evaluated the quantitative results of two blood assays (pp65 antigenemia test and Real-time PCR) and the semi-quantitative results of dried blood sample (DBS) test, obtained in our routine diagnostic activity, in the aim of identifying a valid method for measuring viral load. On completion of the ongoing collection of follow-up data of the studied children, it will be possible to assess its prognostic significance.

## Methods

### Cases

In this study we retrieved and elaborated the results of tests previously performed in our laboratory on blood samples as part of the protocol for cCMV diagnosis. Samples had been collected from 70 consecutive newborns (32 females and 38 males) whose cCMV infection had been diagnosed on the basis of a positive rapid isolation test (SV assay) from urine specimens collected in the first two weeks of life.

Twenty-two newborns (31%) showed symptoms of congenital infection at birth [7]. The other 48 newborns were asymptomatic: 44 of these had been examined having been delivered from mothers with a confirmed primary infection during pregnancy and 2 were identified during a study of newborns from HIV+ mothers, while evidence of exposure to risk factors was not indicated for the 2 remaining newborns.

No data of follow-up are available yet.

Since this was a retrospective and methodological study on the results already available with us, it was not put up for any clearance to an ethical committee. All the patient files are confidential and none could be identified.

### Methods

#### Antigenemia test (pp65)

The leukocyte fraction (PBL) was separated from each blood sample for antigenemia testing in IF assay (*CINA pool pp65, Argene*). The result was expressed as the number of positive cells in 2 × 10^5 ^PBL [8].

#### Real-time Quantitative PCR (qPCR)

CMV-DNA was extracted from 200 μl of separated plasma using the QIAamp DNA Mini kit (QIAGEN) according to the manufacturer's protocol. The amount of viral DNA extracted was then measured in a commercial quantitative PCR assay (Q-CMV Real Time Complete Kit, Nanogen) following the supplier's instructions. The results were expressed as copies/ml.

#### DBS test

Viral DNA in dried blood samples (DBS test) from newborns was tested as described by Binda et al. [9]. CMV DNA was eluted from 3-mm diameter disks, extracted by means of thermal shock and used as the template for a nested PCR specific for a highly conserved region of the CMV gB gene (nt 1942 - nt 2067) [10]. The result was interpreted following an algorithm described by Barbi *et al*. [11], requiring that the test be done on three series of punches.

The positive results were distinguished as:

- "strong" if at least two out of three tests were positive

- "weak" if one out of three tests was confirmed positive in a further series of three.

#### Statistical analysis

The association between DBS test results (weak or strong) and the presence of symptoms was tested using Fisher's exact test, while the Mann-Whitney U test was performed to compare symptomatic and asymptomatic newborn's median number of positive cells in the pp65 antigenemia test, and the median number of CMV DNA copies detected by qPCR. A p-value of 0.05 was considered significant.

## Results and discussion

Results from the blood samples of all 70 neonates were obtained with the DBS test, whilst the presence of inhibitors hampered the assessment of the number of DNA copies by qPCR in the plasma of three children, and the low PBL counts precluded the determination of the pp65 antigenemia in two cases.

The pp65 antigenemia test was positive in 81% (55/68) of samples tested. Compared with children who had asymptomatic infections, those with symptoms had a non-significant, higher degree of positive results (90% vs. 77%; p = 0.3110), and the median value of positive cells was significantly higher (p = 0.0098) (Table 1). Children with neurological symptoms had median values (31 positive cells/2 × 10^5 ^PBL) higher than symptomatic babies with only abnormal biological parameters (2 positive cells/2 × 10^5 ^PBL; p = 0.0218).

**Table 1:** Results of the pp65 (antigenemia), qPCR, and DBS tests in symptomatic and asymptomatic newborns

	***pp65 (no. cells pos/2 × 10***^5^***PBL)***			***qPCR (no. copies/ml)***			***DBS test***		
	
	**Positive (%)**	**Median**	**IQR°**	**Positive (%)**	**Median**	**IQR°**	**Positive (%)**	**Weak**	**Strong**
**Symptomatic**	19/21 (90.5%)	8.0	2-33	22/22 (100.0%)	8,701	1,777-39,945	22/22 (100.0%)	3	19
**Asymptomatic**	36/47 (76.6%)	3.0	2-7	45/45 (100.0%)	5,383	2,359-11,896	48/48 (100.0%)	7	41
	
***p***	0.0098*			0.2193*			0.2858**		

° IQR = interquartile range

* Mann -Whitney test

** Fisher's exact test

**Figure 1:**
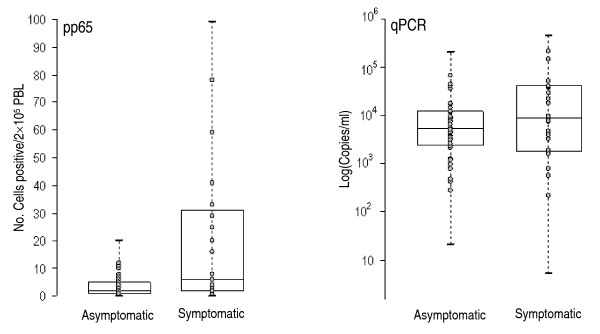
**Number of positive cells/2 × 10^5 ^(pp65) and log_10 _of the number of copies/ml (qPCR)**. The first and third quartiles are the limits of the box, the inner line is the median, and the "whiskers" represent the smallest and largest values. Dots portray single observations.

All 67 plasma samples suitable for qPCR were positive. The order of magnitude of the viral load was 10^3 ^copies/ml in 52% of cases (35/67), of whom 80% were asymptomatic. However, there were no significant differences between the median number of DNA copies/ml in relation to the presence or absence of symptoms (p = 0.22, Table 1) or to the involvement of CNS in symptomatic babies (37,000 vs 7,300 copies/ml; p = 0.0878). As shown in Figure 1, greater variability was detected with the antigenemia test among symptomatic children than in asymptomatic ones, but not with qPCR.

Sixty neonates were strong positives in DBS test whereas the remaining 10 (14%) were weak positives. The DBS test results as with qPCR were not related to the presence or absence of symptoms at birth (p > 0.05; Table 1) nor to the involvement of CNS (p > 0.05). The median number of positive cells in the antigenemia test and copies/ml were significantly higher in children with strong positivity in the DBS test than in those with weakly positive findings (Table 2).

**Table 2:** pp65 antigenemia and qPCR findings in children with either a weakly or strongly positive DBS-test

	Positive DBS test			
	pp65 **(no. cells pos/2 × 10**^5^**PBL)**		qPCR (copies/ml)	
	weak	strong	weak	strong
**Total**	10	58	8	59
**Positive (%)**	7 *(70.0%)*	48 *(82.8%)*	8 *(100%)*	59 *(100%)*
**Median**	1.0	4.5	1,318	7,753
**Interquartile range (IQR)**	1-4	2-11.8	501-3,327	3,136-17,916
*p*	0.0385*		0.0279*	

* Mann -Whitney test

## Conclusions

In this study, two quantitative methods (the pp65 antigenemia test and qPCR) were compared with semi-quantitative DBS test to assess the clinical relevance of hCMV load determination in infected babies both symptomatic and asymptomatic at birth.

In this and a previous study [8], the sensitivity of the antigenemia test to identify an infection was 81% (CI_95%_: 69.3 to 89.1) and 84%, respectively, which was higher than the 42% reported by Revello et al. [3]. This difference might be because of the smaller proportion of symptomatic neonates in the latter study. However all these studies showed significantly higher median counts of pp65 positive PBLs in symptomatic cases. The significant difference in terms of pp65 positive PMN cells, but not as plasma CMV DNA copies/ml, in relation to the clinical situation at birth might be related to the type of sample. Revello [3] and Lanari [5] detected significant differences of viral load. testing fixed amounts of PBLs

Viral DNA was detected by PCR in the acellular blood fraction of all the infants, as observed in other studies [12,13], confirming the higher sensitivity of molecular tests compared with immunological ones.

The quantitative PCR detected a median number of copies/ml of the same order of magnitude (10^3^) as that reported by Halwachs-Baumann et al. [14] even if they tested serum and not plasma. Bradford et al. detected levels that were about one logarithm lower in children with symptomatic infections involving the central nervous system [15]. These viral load values could affect the DBS test findings; in an external quality assessment study, about half the laboratories were unable to detect DNA from blood with a viral load of 10^3 ^copies/ml [16]. Of note, we noticed that there was a loss of titer of about one log between fresh and dried blood samples.

As described in a review by Barbi et al. [11], the DBS test must be repeated on a new series of three samples to confirm a single positivity in about 10% of cases, suggesting that this is indicative of a low viral load in the blood. This was confirmed by the present study, where a lower median level of both pp65-positive cells and DNA copies was detected in weakly positive DBS samples, corresponding to 14% of all those tested. Therefore, some of these cases were probably at the limit of sensitivity and may be missed with DBS testing, particularly if the method employed (sample size, elution, extraction, amplification) and the algorithm for defining positivity are less than optimal. Hence, it is necessary to correctly perform this test and properly interpret the test results.

A comparison of the semi-quantitative measures obtained in DBS test with the quantitative results of real-time PCR on the same samples is ongoing.

Finally, upon completion of clinical and audiological follow-up, the prognostic value of quantitative and semi-quantitative results could be assessed.

## Competing interests

The authors declare that they have no competing interests.

## Authors' contributions

SB, AM, VP, PD, MB participated in the conception and design of study, performed the assays and contributed to the preparation of the manuscript. CC, FM, LP provided clinical data and contributed to draft the manuscript. AB and CR performed the statistical analysis of the results and provided critical comments to improve the manuscript. All authors read and approved the final manuscript.

## Pre-publication history

The pre-publication history for this paper can be accessed here:

http://www.biomedcentral.com/1471-2334/10/24/prepub
